# GATA3 suppresses human fibroblasts-induced metastasis of clear cell renal cell carcinoma via an anti-IL6/STAT3 mechanism

**DOI:** 10.1038/s41417-019-0146-2

**Published:** 2019-10-21

**Authors:** Qianqian Shi, Renfang Xu, Guanglai Song, Hao Lu, Dong Xue, Xiaozhou He, Ying Xia

**Affiliations:** 1 The Third Affiliated Hospital of Soochow University, Changzhou, 213000 China; 2 Shanghai Key Laboratory of Acupuncture Mechanism and Acupoint Function, Fudan University, Shanghai, 200433 China

**Keywords:** Cancer microenvironment, Gene expression, Metastasis

## Abstract

Tumorigenesis and metastasis depend on intricate interactions between genetically altered tumor cells and their surrounding microenvironment. It is, however, unclear regarding the molecular mechanisms underlying the progress and metastasis of human clear-cell renal cell carcinoma in the microenvironment with fibroblasts. In this work, we investigated the effect of normal fibroblasts on the metastasis of renal cancer and the relevant signaling pathways. We isolated normal fibroblasts from normal renal tissues and used normal fibroblast-conditioned medium culture renal cancer cells. The CCK-8 and transwell assays showed that normal fibroblasts conditioned medium significantly enhanced ccRCC cell migration. IL6 mediated the cross talk between normal fibroblasts and the cancer cells, and promoted tumor cell migration through the STAT3 pathway. In contrast, GATA3 was downregulated at both mRNA and protein levels in the normal fibroblast-conditioned medium treated with renal cancer cells, but upregulated in adjacent normal tissues. GATA3 overexpression significantly reduced STAT3 phosphorylation and attenuated the migration in both renal cancer cell and IL6-stimulated renal cancer cell. Taken together, our findings suggest that the IL6/STAT3 pathway plays a crucial role in the normal fibroblast-enhanced clear-cell renal cell carcinoma metastasis, while GATA3 may mitigate this effect by inhibiting IL6/STAT3 signaling.

## Introduction

Renal cell carcinoma (RCC) is the most common type of cancer of the urinary tract, constituting 2–3% of human cancers, and its proportion is increasing worldwide over the past decade [1, 2]. Among them, ccRCC is the most frequent type, accounting for 70–80% of all cases [3]. Treatment for localized RCC is mainly surgical resection. Although it generally has a good prognosis, 25–30% of patients have already developed metastatic disease at the time point of diagnosis and the 5-year survival of advanced RCC is still around only 20% [4]. Thus, discovery of new molecular targets and new treatment strategies for RCC are urgently required.

Tumors consist of cancer cells and stromal cells, including fibroblasts, immune cells, and endothelial cells. In fact, the main cellular constituents of solid tumors are stromal fibroblasts, which are proofed by histological examinations of human tumors [5–7]. Indeed, fibroblasts are important for cancer progression and the most abundant stromal cell type in epithelial tumors [8, 9]. Accumulating evidence has suggested that malignant cells themselves are not sufficient to maintain tumor growth and progression. They are highly dependent on complex dynamic interactions between tumor cells and tumor stroma, mediated by direct cell–cell contact and secreted growth factors and cytokines [10]. Cancer-associated fibroblasts (CAFs) differ from normal fibroblasts (NFs) that are in close contact with tumor cells, in order to adapt to the tumor microenvironment and co-evolve with tumor cells to foster malignancy [11, 12]. Many studies have showed that CAFs isolated from various cancers, including breast, ovarian, and prostate cancers, could promote genomic instability, induce epithelial–mesenchymal transition (EMT), and promote tumor growth and metastasis. These findings confirm that CAFs are intimately involved in almost every step of tumor progression, from initiation to metastasis [13–15]. However, what and how NFs work in the development of renal neoplasms are not well understood yet.

GATA3 is a member of the GATA family of zinc finger nuclear transcription factors, which binds to G-A-T-A nucleotide sequences in the promoter regions of target genes, thereby activating or suppressing the function of these genes [16]. It plays an essential role in the development, proliferation, and differentiation of several types of tissues and cells [17–21]. Initial studies suggested that GATA3 is relatively sensitive and specific for tumors of breast or urothelial origin, and the downregulation of GATA3 has also been found to be associated with a poor prognosis of breast cancer. The significance of this transcription factor, especially for the inhibition of cancer cell metastasis, has been discussed in recent reports [22, 23]. However, how does GATA3 participate in ccRCC development and metastasis is still unknown.

In this work, we explored the influence of normal fibroblasts on the growth and metastasis of ccRCC cells and then conducted mechanistic study on the role of GATA3 in this event. Our novel data suggest that the IL6/ STAT3 signaling pathway enhances the effect of NFs on ccRCC cell metastasis, while GATA3 binds to the −1710~−1530 region of STAT3 promoter and inhibits the IL6-induced STAT3 activation by repressing its transcription.

## Materials and methods

### Isolation and culture of human normal renal fibroblasts

NFs were isolated from human normal renal cortical tissues. These tissues were obtained from five patients who had diagnosed as ccRCC at The Third Affiliated Hospital of Soochow University (Changzhou, Jiangsu, China). These patients had not been treated with chemotherapy before surgery. The collection and the using of specimens were approved by the Institutional Review Board of TMUGH, and the informed consent was obtained from all patients. The specimens were initially soaked in 95% ethanol to protect them from contamination, then washed in phosphate buffered saline (PBS) with 1% penicillin/streptomycin. We used scissors to cut the specimens into sections, which were then digested with collagenase IV (Invitrogen, CA, USA) and 25 mg/mL trypsin at 37 °C for 40 min. Then the mixture was strained through a strainer, and the cells were collected and cultured in the RPIM 1640 containing 20% fetal bovine serum (FBS; Gibco, USA) and 1% penicillin/streptomycin. The culture medium was renewed every 2 days after seeding. The cells were digested with 0.25% trypsin, when cell confluence reached 70%. The digested cells were cultured in the RPIM 1640 with 10% FBS and 1% penicillin/streptomycin.

To prepare the normal fibroblast-conditioned medium (NF-CM), NFs were cultured for 72 h, the conditioned medium (CM) were collected and centrifuged for 10 min at 3000 rpm to remove cell debris.

### Cell lines and reagents

We obtained human clear-cell renal cancer cells (786-O, 769-P, ACHN, and Caki-1) from the American Type Culture Collection. All these cells, except Caki-1, were incubated in the RPIM 1640 (Invitrogen, CA, USA) with 10% FBS and 1% penicillin/streptomycin at 37 °C in a humidified 5% CO_2_ atmosphere, while Caki-1 cells were maintained in 5 A medium (Invitrogen, CA, USA) containing 10% FBS.

Human anti-IL6 antibody was purchased from R&D Systems (Minneapolis, MN, USA). Anti-STAT3, anti-phosphospecific STAT3 (p-STAT3; Tyr705), anti-MMP2, anti-MMP9, anti-GAPDH, anti-E-cadherin, anti-α-SMA antibodies were purchased from Cell Signaling Technology (Beverly, MA, USA).

### Cell proliferation assay

Cells were seeded onto a 96-well plate at a density of 3 × 10^3^. At 24 h post seeding, CM was added and cultured for 3 days, and the cell culture medium (RPIM 1640) was used as control. Cell proliferation was determined by the Cell Counting Kit-8 (Dojindo, Kumamoto, Japan) following the manufacturer’s instruction.

### Transwell assay

Transwell chamber inserts with uncoated matrigel (migration) was used for cell migration assays. The cells (6 × 10^3^ cells) were seeded into the upper chamber of the 24-well culture inserts coated with 200 µL of serum-free medium. In total, 500 µL of complete medium supplemented with 10% FBS was added to the bottom of the inserts, allowing cells to migrate for 48 h. After incubation, the cells on the upper surface of the membrane were removed, whereas those on the lower filter surfaces were fixed with 4% paraformaldehyde for 10 min, stained with crystal violet, and counted in ten random fields from each membrane under a microscope at x200 magnification.

### Quantitative real-time PCR

The total RNA was extracted from cells or tissues using Trizol (Invitrogen, Carlsbad, CA, USA), and cDNA was synthesized using the PrimerScript RT reagent Kit (Takara, Japan). The expression of genes was measured by quantitative PCR (qPCR) using Power SYBR Green Master Mix (ABI, Foster City, CA, USA) on an ABI 7500 real-time system. All primers were designed by Primer 5.0 software and synthesized by SBS Genetech (Beijing, China). We used the following specific primer pairs to quantify the expression of the genes that encode the proteins indicated:

α-SMA-F: CACTGCCGCATCCTCATC

α-SMA-R: TGCTGTTGTAGGTGGTTTCAT

E-cadherin-F: ATTTTTCCCTCGACACCCGAT

E-cadherin-R: TCCCAGGCGTAGACCAAGA

GAPDH-F: GAGAGACCCTCACTGCTG

GAPDH-R: GATGGTACATGACAAGGTGC

GATA3-F: CGAGATGGCACGGGACACTA

GATA3-R: TGGTCTGACAGTTCGCACAGG

The expression levels of genes were calculated based on the cycle threshold (Ct) values. The results were calculated as 2^−ΔΔCt^ by comparing the Ct values of target genes with the Ct values of the reference gene (GAPDH).

### Western blotting

Western blotting was carried out according to the standard protocols. Briefly, harvested cells or tissues were lysed with RIPA buffer containing protease inhibitor (Sigma, Aldrich). The proteins were separated by SDS-PAGE and transferred to a PVDF membrane (Millipore, Bedford, MA, USA). The membranes were blocked with 5% nonfat milk in Tris-buffered saline containing 0.5% Tween-20 (TBS-T) for 1 h at room temperature, then probed with primary antibodies for GAPDH, α-SMA, p-STAT3 (Tyr705), STAT3, E-cadherin, MMP2, MMP9 in TBS-T containing 5% BSA at 4 °C overnight. After washing, the membranes were probed with the HRP-conjugated secondary antibodies. The protein bands were detected with enhanced Chemiluminescence (ECL) (Millipore, Bedford, MA, USA).

### Enzyme-linked immunosorbent assay

Renal cancer cells were cultured with or without NF-CM for indicated times, the supernatants were collected for IL6, VEGF, CXCL12, IL1β detection using human ELISA kits (eBioscience, San Diego, CA, USA), according to the manufacturer’s instructions. Culture supernatants were diluted 10 (VEGF, CXCL12, IL1β) or 20 (IL6). Briefly, immunoassay plates were precoated with the capture antibody at 4 °C overnight. After blocked for 1 h, supernatants or standard factor were added and incubated for 2 h. The plates were washed and incubated with biotin-labeled factor antibody for 2 h, then with streptavidin–HRP complex. After 30 min incubation, tetramethylbenzidine (TMB) color-substrate solution was added and incubated for 15 min The plates were read on a microplate reader (BioTek Instruments Inc., Winooski, VT, USA) at 450 nm. Levels of each growth factor were assayed in three independent sets of each type of conditioned medium, and normalized to the total cellular protein content.

### Immunofluorescence

Cells were seeded on the cover-slips in 24-well plates and cultured overnight, then fixed in 4% paraformaldehyde for 20 min. After washing with PBS, cells were permeabilised in 0.25% Triton X-100 for 15 min, washed with PBS, and blocked with 5% BSA for 60 min. Then cells were incubated with α-SMA antibody (Abcam, USA; 1:100), E-cadherin antibody (Sigma, USA; 1:100) overnight at 4 °C. After washing with PBS, cells were incubated with an Alexa Fluor 488-conjugated anti-mouse IgG F(abʹ)2 fragment (Invitrogen, USA; 1:200) or an Alexa Fluor 549-conjugated anti-mouse IgG F(abʹ)2 fragment (Invitrogen, USA; 1:200) at room temperature in the dark. The cells were observed using a fluorescence microscope (Nikon, Tokyo, Japan).

### Clinical tissue samples

Thirty pairs of ccRCC and adjacent normal tissues were collected from patients who underwent curative surgery without radiotherapy or chemotherapy at the Department of Urinary Surgery, The Third Affiliated Hospital of Soochow University (Jiangsu, China) between 2012 and 2017. The pathological nature was confirmed by examination with hematoxylin and eosin staining. Each pair of ccRCC and adjacent tissue were stored at −80 °C before use. This study was approved by the Ethics Committee of The Third Affiliated Hospital of Soochow University. Informed consent was obtained from all the patients.

### Cell transfection

Human ccRCC cells (786-O, 769-P, ACHN, and Caki-1), were transfected with vectors or GATA3 overexpression plasmid according to the standard protocols.

### Statistical analysis

All experiments were repeated in independent sets of work for at least three times (*n* = 3 or more). The results are presented as mean ± standard deviation (SD). Groups and among-group comparisons were conducted using the Student’s *t* test and analysis of variance, respectively. Differences were regarded as statistically significant when the *P*-value was <0.05.

## Results

### A-SMA and vimentin were expressed in the NFs isolated from different patients

**Fig. 1:**
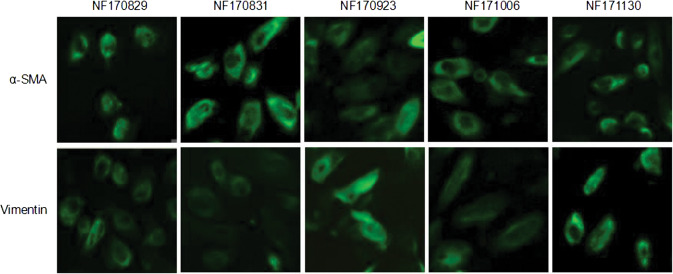
Identification of the isolated fibroblasts. Five normal fibroblasts (NFs) named as NF170829, NF170831, NF170923, NF171006, and NF171130 were isolated from the normal renal cortical tissues of five different patients. Note that all these cells were positive for α-SMA and vimentin staining in cytoplasm by immunofluorescent examinations

We isolated NFs from five normal renal cortical tissues (>6 cm apart from renal cancer tissues) named as NF170829, NF170831, NF170923, NF171006, NF171130, respectively. After passaging the primary cultured cells, the cells displayed a thin and spindle-like appearance. In order to identify whether the cultivated cells were NFs, we examined the expressions of fibroblasts markers α-SMA and vimentin by immunofluorescent staining. As shown in Fig. 1, all the cells were positive for α-SMA and vimentin, suggesting that all these isolated cells were NFs.

### NFs significantly weakened the proliferation, but enhanced the migration of ccRCC cells with no effect on EMT

**Fig. 2:**
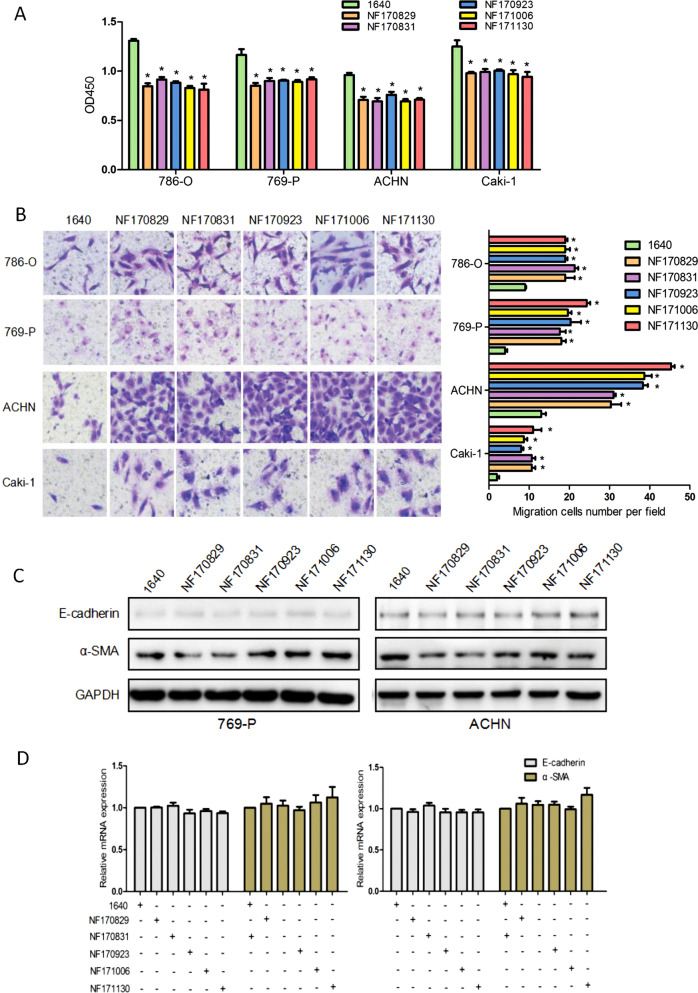
NFs decreased ccRCC cells proliferation and increased migration without EMT. **a** CcRCC cells were cultured for 72 h in the presence or absence of NFs (NF170829, NF170831, NF170923, NF171006, NF171130, respectively) conditioned media. CCK-8 test was performed to evaluate the proliferation ability of different ccRCC cells (786-O, 769-P, ACHN, Caki-1). *P < 0.05 vs. 1640. **b** Transwell assays were carried out to detect cells migration ability. Representative photographs are presented in ×200 magnification. *P < 0.05 vs. 1640. **c** CcRCC cells were cultured for 24 h in the presence or absence of five different NF-CMs, respectively. Several markers associated with the EMT process, such as α-SMA and vimentin were detected by western blotting analysis. **d** The mRNA expression of α-SMA and vimentin were detected by qRT-PCR. All results did not have any statistical significance. Note that the addition of the NF-CM weakened the proliferation and enhanced the migration of the ccRCC cells, but (the addition of the NF-CM) did not induce EMT

To determine whether NFs have a significant impact on the proliferation and migration of renal cancer cells, we examined 786-O, 769-P, ACHN, and Caki-1 cells in the presence or absence of NF-CM. NF-CM was collected from RPIM 1640 culturing NFs for 72 h. We first evaluated the effect of different NF-CMs (NF170829, NF170831, NF170923, NF171006, and NF171130) on the growth of ccRCC cells by CCK-8 assay. Figure 2a showed that five different NF-CMs attenuated ccRCC cells proliferation to a significantly greater degree than control 1640 medium (*P* < 0.05). The effects of NF-CMs on cell migration were examined by transwell assay. As shown in Fig. 2b, all NF-CMs largely boosted the migration ability of ccRCC cells as compared with the control 1640 medium (*P* < 0.05).

Some previous studies suggest that NFs can stimulate EMT, which confers metastatic and self-renewal potential to cancer cells during tumorigenesis. However, we did not found any appreciable influence of NFs on the EMT of ccRCC cells. We treated ccRCC cells with five different NF-CMs and detected the expression of epithelial marker E-cadherin and mesenchymal marker α-SMA using western blotting and qRT-PCR. All the ccRCC cells cultivated with these NF-CMs had no significant changes in protein levels of E-cadherin and α-SMA (Fig. 2c), which was further confirmed at the mRNA level through our quantitative PCR assays (Fig. 2d).

Taken together, these results demonstrate that NFs can attenuate the proliferation and enhance the migration potential of renal cancer cells with no appreciable effect on EMT programming.

### NF-CM promoted IL6 secretion and activated STAT3 signaling in ccRCC cells

**Fig. 3:**
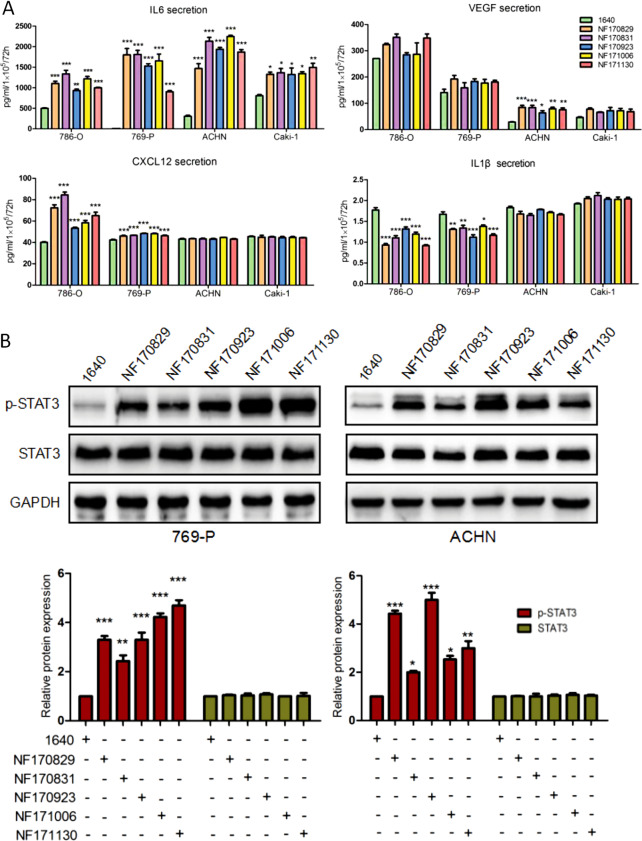
The cytokines secretion and STAT3 activation after the co-culture of NF-CM and ccRCC cells. CcRCC cells were cultured for 72 h in the presence or absence of different NF-CMs. **a** Expression levels of IL6, VEGF, IL1β, and CXCL12 in media were measured using ELISA, respectively. IL6 levels were remarkably increased in all cells, while VEGF was only increased in ACHN cells. CXCL12 secretion was increased in 786-O and 769-P cells, but IL1β secretion was decreased. *P < 0.05, **P < 0.01, ***P < 0.005 vs. 1640. **b** Protein expression of p-STAT3 and total STAT3 were detected by western blotting, and representative results from one of the three independent experiments are presented. *P < 0.05, **P < 0.01, ***P < 0.001 vs. 1640. Note that NF-CM induced expression of IL6, VEGF and CXCL12 increased, decreased that of IL1β and activated STAT3 in ccRCC cells

To address the effects of NFs on cytokines potentially involved in renal cancer progression, we examined, with ELISA assay, some relevant cytokines, such as IL6, VEGF, CXCL12, and IL1β, in medium with the presence or absence of NF-CM after 72 h. Figure 3a shows that IL6 secretion was significantly increased in all ccRCC cells culturing with five NF-CMs, especially 769-P and ACHN cells. In fact, IL6 was upregulated by more than four fold (*P* < 0.001) in 769-P and ACHN cells. In contrast, CXCL12 was increased by only one fold (*P* < 0.001) in 786-O and 769-P cells, and VEGF was statistically increased two fold (*P* < 0.001) only in ACHN cells. On the opposite side, the secretion of IL1β was decreased to 60% (*P* < 0.001) and 75% (*P* < 0.001) in 786-O and 769-P cells, comparing with the control (the RPIM 1640 medium). These data suggest that IL6 is a major player in all ccRCC cells in response to NF-CM stimulation.

Furthermore, we therefore investigated the effect of NFs on STAT3 signaling. As shown in Fig. 3b, the phosphorylated STAT3 (p-STAT3) was increased by onefold to fourfold (*P* < 0.001) in 769-P and ACHN cells with the presence of NF-CM, while total STAT3 level had no changes at all.

These results suggest that NFs may stimulate renal cancer cells to secret IL6 and activate the STAT3 signaling pathway.

### The IL6/STAT3 pathway mediated the effect of NFs on ccRCC cell migration

**Fig. 4:**
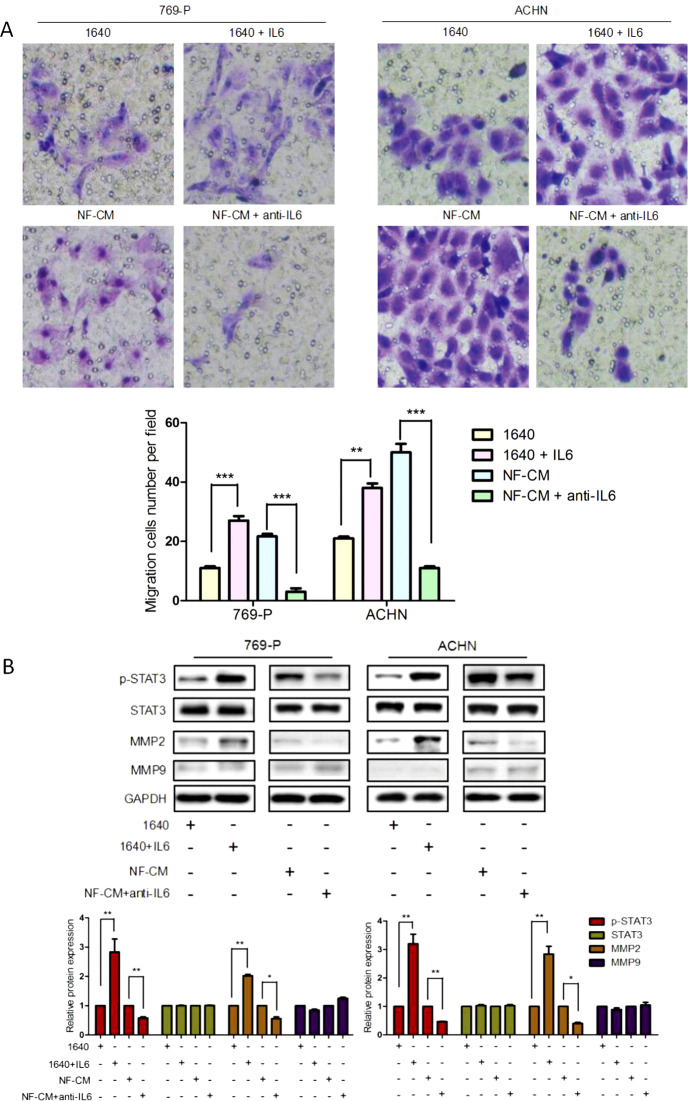
The effects of IL6 on ccRCC cells migration and the IL6/STAT3 signaling pathway. CcRCC cells (769-P and ACHN) were treated with 1640 or 1640 + IL6 (200 μg/ml), NF-CM or NF-CM + anti-IL6 (50 μg/ml) for 48 h. **a** Cells migration ability was measured by transwell assays. Migration cells at 48-h time point were presented. ***P < 0.001 vs. 1640. Anti-IL6 antibody (50 μg/ml) inhibited the effect. **P < 0.01, ***P < 0.001 vs. NF-CM. **b** P-STAT3, STAT3, MMP2, and MMP9 were analyzed using western blotting. Values represent the mean ± SD from three independent experiments. *P < 0.05, **P < 0.01 1640 vs. 1640 + IL6, NF-CM vs. NF-CM + anti-IL6. Note that recombinant human IL6 could promote ccRCC cells migration and activate STAT3 and MMP2, while anti-IL6 antibody could abolish the effect

To further elucidate the role of the IL6/STAT3 pathway in the NF-promoted ccRCC cell migration, we added 200 µg/ml of human recombinant IL6 to normal medium cultured 769-P and ACHN cells, or 50 μg/ml of IL6 neutralizing antibody (anti-IL6 antibody) to NF-CM cultured 769-P and ACHN cells, and the performed transwell assay to evaluate ccRCC cells migration. Compared with the control, IL6 addition largely enhanced the ccRCC cell migration (*P* < 0.005), especially in 769-P cells (*P* < 0.001) (Fig. 4a). This result was further conformed by the results of anti-IL6 antibody treatment. As shown in Fig. 4a, anti-IL6 antibody almost abolished the effect of NF-CM on the cells migration (*P* < 0.001).

Next, we investigated the effect of IL6 on STAT3 activation and metastasis-related genes MMP2 and MMP9. Western blotting results (Fig. 4b) showed that IL6 addition dramatically increased the level of p-STAT3 by more than two fold (*P* < 0.005) and MMP2 more than one fold (*P* < 0.005) at the protein level, while it had no effect on MMP9. Moreover, anti-IL6 blocked the NF-CM induced increase in p-STAT3 (*P* < 0.005) and MMP2 (*P* < 0.05), which could be effectively blocked by the addition of anti-IL6 antibody, suggesting that IL6 is crucial to this process. Collectively, these results indicate that the IL6-increased STAT3 activation and MMP2 expression in renal cancer cells can be antagonized by concomitant administration of anti-IL6 antibody.

### GATA3 is downregulated in ccRCC tissues and cells

**Fig. 5:**
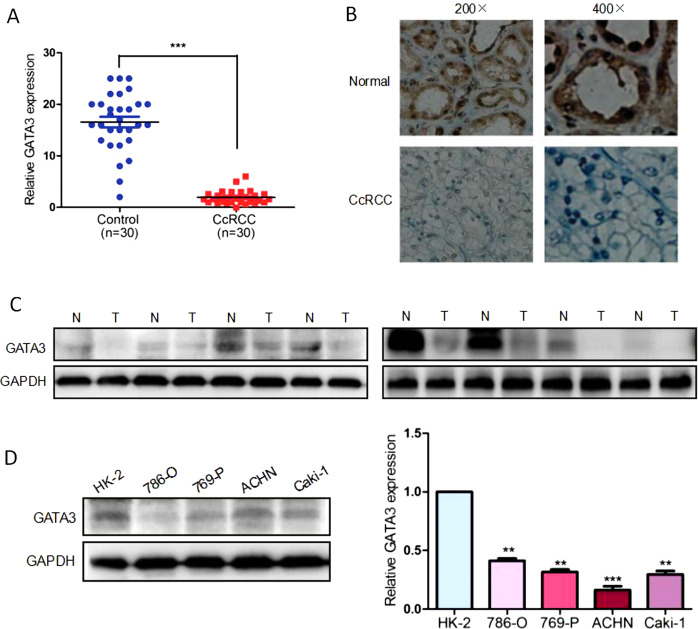
Downregulation of GATA3 in ccRCC tissues. **a** The total mRNA expression levels of GATA3 were examined by qRT-PCR in 30 pairs of normal and ccRCC tissues. Each dot in the graph represents one sample. *P < 0.05 vs. normal tissues. **b** Immunohistochemical analysis of GATA3 protein expression in ccRCC tissues and matched adjacent normal tissues. Higher staining of GATA3 was observed in normal renal tissues. **c** Whole protein was lysed from ten pairs of normal and ccRCC tissues. Western blot was performed to determine the relative protein level of GATA3. GAPDH was used as a control. The immunoblot shown is a representative image. N, paired normal tissue; T, tumor tissue. ***P < 0.001 vs. control. **d** The protein expression levels of GATA3 in HK-2, 768-O, 769-P, ACHN, and Caki-1 cells. **P < 0.005, ***P < 0.001 vs. HK-2 cells. Note that GATA3 was downregulated in the ccRCC tissues and cells

To investigate the GATA3 expression in ccRCC, we collected 30 pairs of tumor tissue samples and adjacent normal tissue samples from ccRCC patients. The expression of GATA3 was detected by qRT-PCR and western blotting. As shown in Figs 5a, c, both mRNA (*P* < 0.001) and protein levels of GATA3 were significantly downregulated in tumor tissue samples. Interestingly, marked overexpression of the GATA3 protein, as compared with the ccRCC tissues, was detected in the normal tissue specimens by IHC (Fig. 5b). In agreement with the results obtained in the above tissues, GATA3 was also downregulated dramatically in several ccRCC cell lines, such as 786-O, 769-P, ACHN, and Caki-1 in comparison with normal human renal cell line HK-2. As shown in Fig. 5d, in 786-O, 769-P, ACHN, and Caki-1 cells, GATA3 was decreased to 45% (*P* < 0.005), 40% (*P* < 0.005), 20% (*P* < 0.001), and 39% (*P* < 0.005) in comparison with HK-2 cells, respectively.

### Overexpression of GATA3 suppressed ccRCC cell migration

**Fig. 6:**
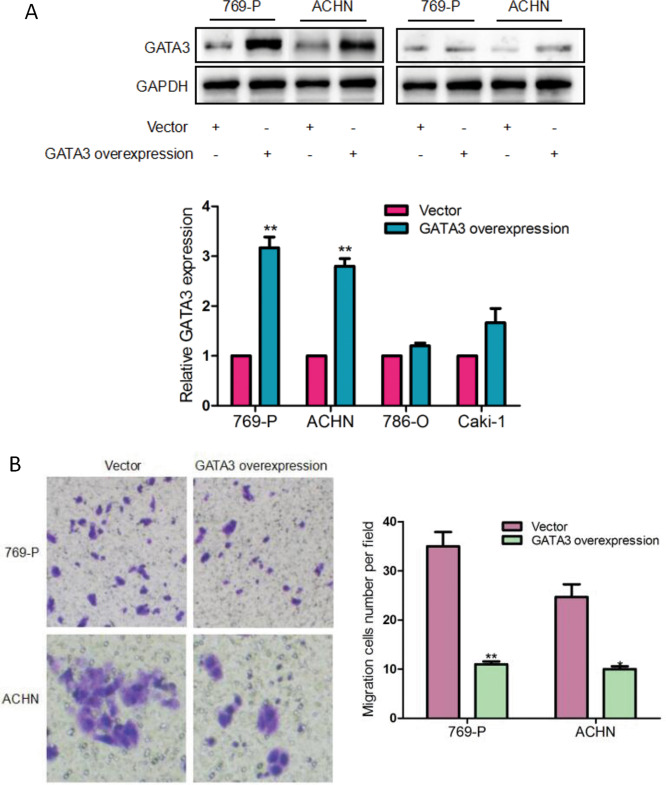
The effect of GATA3 on ccRCC cells migration. **a** CcRCC cells (786-O, 769-P, ACHN, Caki-1) were transfected with vector or overexpression GATA3 plasmid for 48 h, the expression of GATA3 was measured by western blotting. **P < 0.01 vs. vector. **b** GATA3 was overexpressed in 769-P and ACHN cells. Transwell assay was used to detect the effect of GATA3 on cells migration. *P < 0.05, **P < 0.01 vs. vector. Note that restoration of GATA3 inhibited ccRCC cells migration

To explore the effect of GATA3 on the migration of ccRCC cells, four different ccRCC cell lines, i.e., 786-O, 769-P, ACHN, and Caki-1 cells, were transfected with GATA3 overexpression plasmid or control vector, respectively, and then the cell migration ability was determined by transwell assay. As seen in Fig. 6a, GATA3 protein expression was markedly raised by 1–2-fold in 769-P/GATA3 and ACHN/GATA3 cells (769-P and ACHN cells transfected with GATA3 overexpression plasmid) (*P* < 0.005) when compared with that of 769-P/vector and ACHN/vector (the cells transfected with vector) cells. In sharp contrast to the control group, the transwell results (Fig. 6b) showed that migratory behavior of 769-P/GATA3 and ACHN/GATA3 cells was largely reduced to 25% (*P* < 0.005) and 45% (*P* < 0.05), respectively.

### GATA3 inhibits the activation of STAT3 induced by IL6 in ccRCC cells

**Fig. 7:**
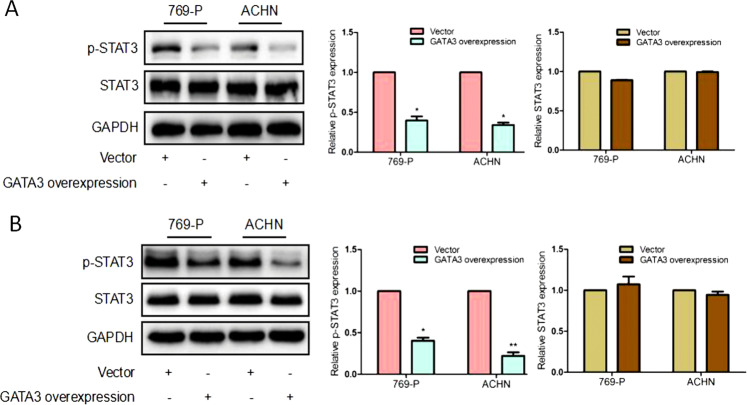
GATA3-induced attenuation of p-STAT3 activation in ccRCC cells. The 769-P and ACHN cells were transfected with overexpression GATA3 plasmid or control vector for 48 h, and (**a**) the relative protein expressions of p-STAT3 and STAT3 were detected by western blotting. *P < 0.05 vs. vector. **b** 769-P/vector, 769-P/GATA3, and ACHN/vector ACHN/GATA3 were incubated with 200 μg/ml IL-6 for 48 h. The expression of p-STAT3 and STAT3 was detected by western blotting. *P < 0.05, **P < 0.001 vs. vector. The representative results from one of the three independent experiments are presented. Note that GATA3 inhibited the IL6-induced STAT3 activation

Since GATA3 bound to the −1710~−1530 region of STAT3 promoter and repressed its transcription [24], we determined whether GATA3 regulated STAT3 activation in ccRCC cells. Figure 7a showed that the levels of p-STAT3 in 769-P/GATA3 and ACHN/GATA3 cells were significantly decreased to 47% (*P* < 0.05) and 45% (*P* < 0.05) of the control levels when compared with those of 769-P/vector and ACHN/vector cells.

Furthermore, we explored the influence of GATA3 on IL6-mediated activation of STAT3. We incubated 796-P/vector, 769-P/GATA3, ACHN/vector, and ACHN/GATA3 cells with 200 μg/ml IL-6 alone for 48 h, and then detected the levels of p-STAT3 in these cells. As shown in Fig. 7b, the level of p-STAT3 was attenuated in 769-P/GATA3 (*P* < 0.05) and ACHN/GATA3 cells (*P* < 0.01).

These results suggest that GATA3 can significantly inhibit the IL6-induced STAT3 activation.

## Discussion

We have made two novel findings in this study: (1) NFs largely enhanced ccRCC cell migration through the IL6/STAT3 pathway and (2) GATA3 suppressed such migration by inhibiting IL6-induced activation of STAT3.

Renal cancer is one of the most common and malignant types of cancer with a rapid progression and distant metastasis. It is generally recognized that cancer metastasis is not merely a local problem and it is also controlled by the tumor microenvironment (TME) [25–27], in which fibroblasts are the main component and play extensive roles in regulating multiple tumor metastases, including gastric cancer [28], lung cancer [29], and breast cancer [30]. This study provides the first evidence regarding the effect of normal fibroblasts on renal cancer cells and shows that IL6 can promotes the migration of all four renal cancer cells tested (786-O, 769-P, ACHN, and Caki-1), suggesting an increased ability of metastasis.

EMT has been previously proposed to be essential for tumor metastasis [31–34], and CAFs can induce EMT and promote cancer cell motility in gastric cancer [35], breast cancers [36], and pancreatic cancer [37]. However, some investigators have recently challenged the conceptual framework that EMT promotes metastasis, and demonstrated that EMT was dispensable for metastasis [38–41]. Moreover, it is unclear whether NF-CM affects renal cancer cell metastasis through EMT. We therefore detected epithelial marker E-cadherin and mesenchymal marker α-SMA in this study to address this important issue. Our result showed that NF-CM did not change the expressions of E-cadherin and α-SMA, suggesting that EMT might not happen in the process of the NF-CM enhanced metastasis in renal cancer cells.

Tumor metastasis is usually associated with abnormal expression of cytokines, especially CXCL12 [42–46], VEGF [47, 48], IL1β [49, 50], and IL6 [51, 52], while CAFs can promote tumor growth and invasion via a plethora of active factors [53]. We therefore checked these specific cytokines in different cancer cells with the presence or absence of different NF-CMs. Interestingly, we found that IL6 was enormously increased in all renal cancer cells, while other three cytokines only slightly increased or decreased in one/two kinds of the renal cancer cells. Furthermore, we found that IL6 could markedly stimulate 769-P and ACHN cell migration, while the anti-IL6 antibody could neutralize such effect. Therefore, it is likely that IL6 plays an important role in renal cancer metastasis as in the lungs, in which IL6 promotes lung adenocarcinoma progression and metastasis. Since several lines of evidence [54–56] have shown that IL6 and STAT are involved in tumor metastasis in some types of cancers, we asked if IL6 relies on STAT for renal cell metastasis. We chose 769-P and ACHN cells to examine the expression of phosphorylated STAT3 and metastasis-related genes MMP2 and MMP9 and found that IL6 increased the activation of STAT3 and MMP2 in both cell types, and the IL6 effect was partially attenuated by the anti-IL6 antibody, implying that the IL6/STAT3/MMP2 pathway plays a significant role in the promotion of metastasis in renal cancer.

**Fig. 8:**
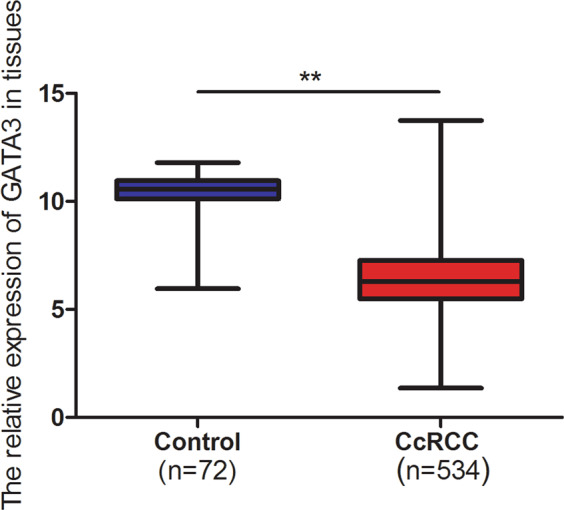
GATA3 expression in TCGA database. GATA3 was decreased in 534 ccRCC tissues, compared with 72 normal tissues in TCGA database

GATA3 has been reported as a sensitive and specific marker for urothelial and breast carcinomas in tissue sections [53]. However, it may have differential impacts on different organs [57]. In fact, it remains unclear regarding its role in renal carcinoma. We analyzed the differential gene expression between ccRCC tissues and normal tissues in three Gene Expression Omnibus (GEO) datasets (GSE16449, GSE53757 and GSE66272) (http://www.ncbi.nlm.nih.gov/geo) and The Cancer Genome Atlas (TCGA) (https://cancergenome.nih.gov/) database (534 tumor tissues and 72 normal tissues). The results show that GATA3 was remarkably decreased in ccRCC tissues (Fig. 8). Moreover, our explorations into TCGA database further suggest the relevance of GATA3 expression to the tumor size, distant metastasis, and pathologic stage in ccRCC patients (Table 1). Taken together, our present study renders us confident for that GATA3 functions to inhibit IL6-induced STAT3 activation in the microenvironment with fibroblasts. GATA3 is a strong inhibitor of ccRCC metastasis, because GATA3 was largely downregulated in the ccRCC tissues and cells, while the overexpression of GATA3 suppressed ccRCC cell migration.

**Table 1:** GATA3 expression and clinical characteristics of ccRCC

Variables	Number (total *n* = 534)	GATA3 expression		*P-*value
		Low	High	
Age (yr)				0.3867
≤60	264 (49.44%)	137	127	
>60	270 (50.56%)	130	140	
Gender				0.1029
Male	346 (64.79%)	164	182	
Female	188 (35.21%)	103	85	
Tumor size				0.0122*
T1	273 (51.12%)	153	120	
T2	69 (12.92%)	35	34	
T3	181 (33.90%)	76	105	
T4	11 (2.06%)	3	8	
Lymph nodes metastasis				0.8696*
N0	240 (44.94%)	117	123	
N1	16 (3.00%)	8	8	
N/A	278 (52.06%)	142	136	
Distant metastasis				0.0112*
M0	424 (79.40%)	212	212	
M1	80 (14.98%)	33	47	
N/A	30 (5.62%)	22	8	
Stage				0.0204*
I	269 (50.38%)	151	118	
II	57 (10.67%)	29	28	
III	123 (23.03%)	50	73	
IV	85 (15.92%)	37	48	

*N/A* not available

All data of the 534 patients with ccRCC were collected from TCGA database. Low GATA3 expression: lower than the median level of GATA3 expression of total patients. High GATA3 expression: higher than the median level of GATA3 expression of total patients. The patients’ number in each district was counted and analyzed by chi-square test. The statistical results suggest that the expression of GATA3 was significantly correlated with the tumor size, distant metastasis, and pathologic stage. However, there is no significant association between GATA3 expression and patients’ age, gender, and lymph nodes metastasis

*P < 0.05

**Fig. 9:**
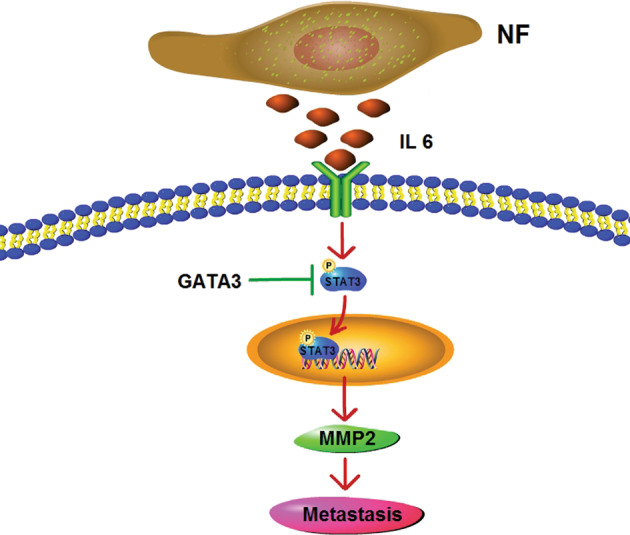
The schematic interaction of GATA3 and IL6 in the STAT3 activation and ccRCC cell metastasis

In conclusion, our new findings clarify a fundamental mechanism for renal cancer cell metastasis. NFs have a profound impact on ccRCC migration and accelerate the cancer invasion and metastasis, which was mainly through a IL6-induced STAT3 activation. On the other side, GATA3 serves as a strong inhibitor of STAT3 activation and thus attenuates the metastasis. Their mechanistic interaction is schematically illustrated in Fig. 9. Taken together, inhibiting the IL6/STAT3 signaling pathway and promoting GATA3 signals may be a therapeutic intervention for the treatment of renal cancer cell metastasis.

## Supplementary information

GATA3 expression and clinical characteristics of ccRCC
