# Correction: GATA3 suppresses human fibroblasts-induced metastasis of clear cell renal cell carcinoma via an anti-IL6/STAT3 mechanism

**DOI:** 10.1038/s41417-020-00229-w

**Published:** 2020-11-06

**Authors:** Qianqian Shi, Renfang Xu, Guanglai Song, Hao Lu, Dong Xue, Xiaozhou He, Ying Xia

**Affiliations:** 1 The Third Affiliated Hospital of Soochow University, Changzhou, 213000 China; 2 Shanghai Key Laboratory of Acupuncture Mechanism and Acupoint Function, Fudan University, Shanghai, 200433 China

**Keywords:** Cancer microenvironment, Gene expression, Metastasis

Correction to: *Cancer Gene Therapy*

10.1038/s41417-019-0146-2

The original version of this Article contained misplaced/mislabeled images in Figs. 2b, 6a and 7b. All these figures have been corrected in both the PDF and HTML versions of the Article.

**Figure:**
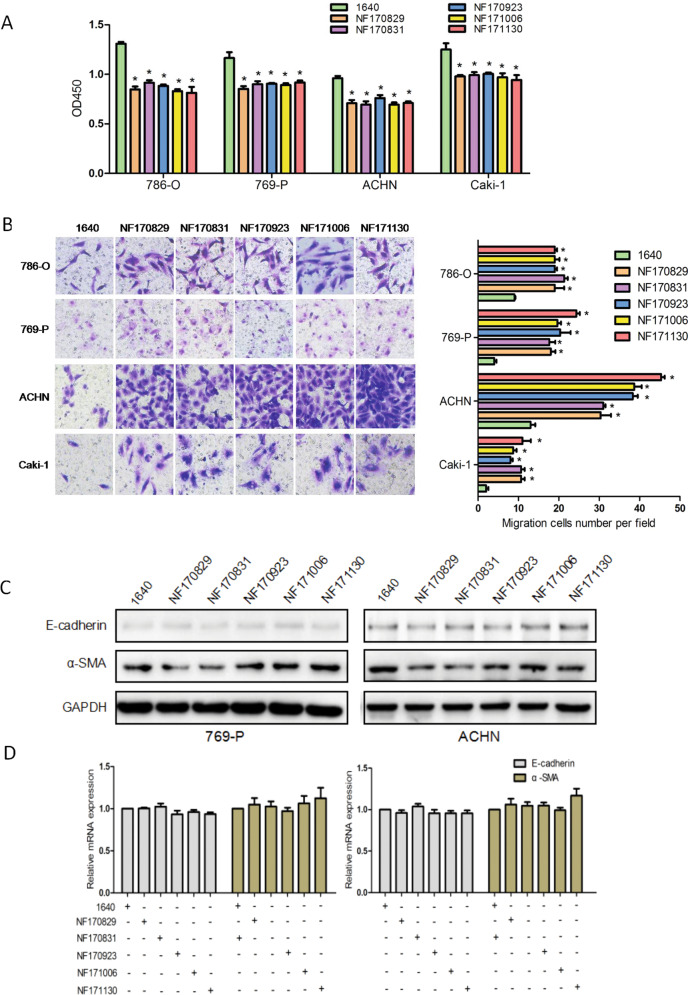
Fig. 2.

**Figure:**
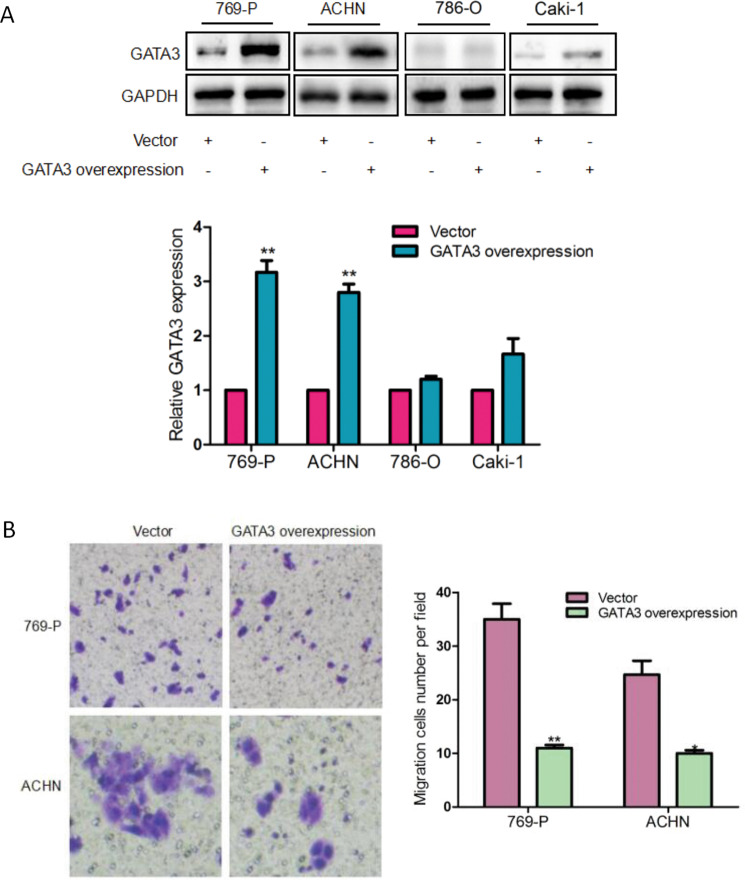
Fig. 6.

**Figure:**
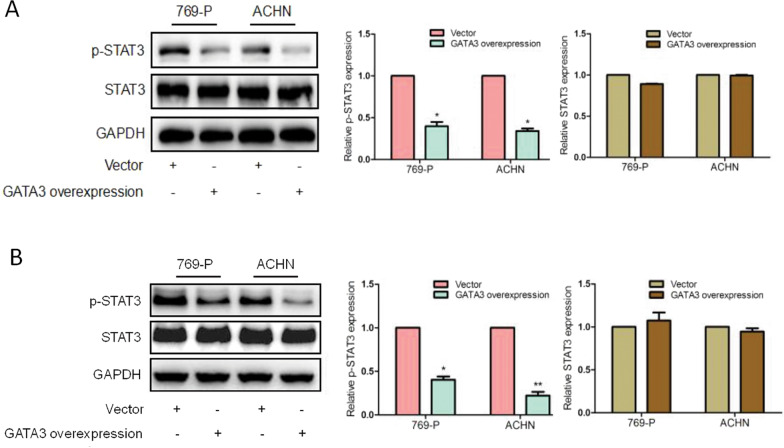
Fig. 7.

Figure 2b: The wrong images were placed in the original when choosing representative images from the countless image data. The figure has now been corrected.

Figure 6a: Parts of the image were labelled incorrectly. The correct group names should be “769-P, ACHN, 786-O and Caki-1” as stated in the legend of Fig. 6. Moreover, we have separated these four groups of images to avoid potentially misleading readers, since there was no comparison among the data of these groups.

Figure 7b: Mismatched total STAT3 and GAPDH images. Although they had no appreciable changes in all groups and nobody may notice it, we feel that the correction will be better in terms of scientific spirit.

The amendments to the above mentioned figures do not affect the statistical analysis and conclusions of the article. For example, in Fig. 6a, GATA3 protein density of ACHN largely increased in the cells after the transfection with the GATA3 overexpression plasmid, while GAPDH signal bands had no appreciable changes. We have provided the original and uncropped images from three different experiments strongly support this conclusion.

