# Supplementary material for: GATA3 suppresses human fibroblasts-induced metastasis of clear cell renal cell carcinoma via an anti-IL6/STAT3 mechanism
Source: Cancer Gene Ther. 2019 Oct 21;27(9):726–38. doi: 10.1038/s41417-019-0146-2 (PMC7492134; doi:10.1038/s41417-019-0146-2)
Supplement: Supplementary file 1 — GATA3 expression and clinical characteristics of ccRCC [file 41417_2019_146_MOESM1_ESM.doc]

| **Variables** | **Number**  **(Total n=534)** | **GATA3 expression** | | ***P-*value** |
| --- | --- | --- | --- | --- |
|  |  | **Low** | **High** |  |
| **Age (yr)** |  |  |  | *P*=0.3867 |
| ≤60 | 264 (49.44%) | 137 | 127 |  |
| >60 | 270 (50.56%) | 130 | 140 |  |
| **Gender** |  |  |  | *P*=0.1029 |
| male | 346 (64.79%) | 164 | 182 |  |
| female | 188 (35.21%) | 103 | 85 |  |
| **Tumor size** |  |  |  | **P*=0.0122 |
| T1 | 273 (51.12%) | 153 | 120 |  |
| T2 | 69 (12.92%) | 35 | 34 |  |
| T3 | 181 (33.90%) | 76 | 105 |  |
| T4 | 11 (2.06%) | 3 | 8 |  |
| **Lymph nodes metastasis** |  |  |  | *P*=0.8696 |
| N0 | 240 (44.94%) | 117 | 123 |  |
| N1 | 16 (3.00%) | 8 | 8 |  |
| N/A | 278 (52.06%) | 142 | 136 |  |
| **Distant metastasis** |  |  |  | **P*=0.0112 |
| M0 | 424 (79.40%) | 212 | 212 |  |
| M1 | 80 (14.98%) | 33 | 47 |  |
| N/A | 30 (5.62%) | 22 | 8 |  |
| **Stage** |  |  |  | **P*=0.0204 |
| I | 269 (50.38%) | 151 | 118 |  |
| II | 57 (10.67%) | 29 | 28 |  |
| III | 123 (23.03%) | 50 | 73 |  |
| IV | 85 (15.92%) | 37 | 48 |  |
| N/A: not available | | | | |

**Table 1.** **GATA3 expression and clinical characteristics of ccRCC**

All data of the 534 patients with ccRCC were collected from TCGA database. Low GATA3 expression: lower than the median level of GATA3 expression of total patients. High GATA3 expression: higher than the median level of GATA3 expression of total patients. The patients’ number in each district was counted and analyzed by chi-square test. The statistical results suggest that the expression of GATA3 was significantly correlated with the tumor size, distant metastasis and pathologic stage. However, there is no significant association between GATA3 expression and patients’ age, gender and lymph nodes metastasis.
